# Evaluation of the Epitogen Lyme Detect IgG ELISA: a novel peptide multiplexing approach

**DOI:** 10.1128/spectrum.01675-24

**Published:** 2024-10-22

**Authors:** Tiehui Wang, Alex Wang, Rodanthi Zindrili, Elena Melis, Swapna Guntupalli, Robin Brittain-Long, Mirela Delibegovic, Christopher J. Secombes, Nimesh Mody, Sally Mavin, Ralfs Buks

**Affiliations:** 1 EpitogenX Ltd, Foresterhill Health Campus, Aberdeen, United Kingdom; 2 University of Aberdeen, Aberdeen, United Kingdom; 3 Scottish Lyme Disease and Tick-Borne Infections Reference Laboratory, Raigmore Hospital, Inverness, United Kingdom; 4 Department of Infectious Diseases, NHS Grampian, Aberdeen Royal Infirmary, Aberdeen, United Kingdom; 5 Aberdeen Cardiovascular and Diabetes Centre, Institute of Medical Sciences, University of Aberdeen, Aberdeen, United Kingdom; National Microbiology Laboratory, Winnipeg, Manitoba, Canada

**Keywords:** Lyme borreliosis, diagnostics, validation, immunodominance, peptide multiplex

## Abstract

Lyme Borreliosis (LB), or Lyme disease, is a growing health concern caused by *Borrelia burgdorferi sensu lato* (Bbsl) bacteria transmitted through tick bites, and untreated cases can lead to severe health complications. Existing serology tests, while valuable, have low sensitivity in early infection stages where diagnosis is vital, interpretation variability, and false positives from cross-reactivity, while direct detection methods also suffer from low sensitivity, due to the inconsistent presence of Bbsl components in clinical samples. This study validated the diagnostic performance of the novel Epitogen Lyme Detect IgG enzyme-linked immunosorbent assay (ELISA) based on scaffold-displayed peptide antigens, using 120 specific immunodominant epitopes selected from 37 antigenic bacterial proteins corresponding to the main pathogenic Bbsl genospecies. Using 220 serum samples from Scottish patients with early, late, and disseminated LB, the assay’s sensitivity was compared with that of the LIAISON Borrelia IgG CLIA, while specificity was assessed with 198 control samples, including healthy individuals and patients with diseases that are humorally similar. The Epitogen Lyme Detect IgG assay demonstrated comparable performance to the LIAISON Borrelia IgG in disseminated and late LB (Lyme neuroborreliosis, acrodermatitis chronica atrophicans, and Lyme arthritis). Notably, the Epitogen Lyme Detect IgG showed significantly higher sensitivity in patients with suspected erythema migrans, while maintaining high specificity. The Epitogen Lyme Detect IgG ELISA offers a promising advancement in LB diagnostics, demonstrating its potential for more accurate and timely diagnosis, particularly in the early stages of LB infection.

**IMPORTANCE:**

Lyme Borreliosis (LB), caused by *Borrelia burgdorferi sensu lato* bacteria, poses significant health risks if undiagnosed or diagnosed late. Current diagnostic tests have limitations, especially in early-stage detection. This study validates the Epitogen Lyme Detect IgG enzyme-linked immunosorbent assay, demonstrating superior sensitivity in early LB detection while maintaining high specificity. The Epitogen Lyme Detect IgG comprises a suite of 120 immunodominant IgG epitopes/peptides from 37 bacterial antigens, covering the main LB-causing species: *Borrelia burgdorferi sensu stricto*, *Borrelia afzelii*, *Borrelia garinii*, and *Borrelia mayonii*. The novel design of multiplexing peptide antigens onto a scaffold to facilitate expression, correct folding, and orientation of the relevant peptides offers a promising advancement, potentially leading to more accurate and timely LB diagnoses and improving patient outcomes.

## INTRODUCTION

Lyme borreliosis (LB), commonly known as Lyme disease, is the most prevalent vector-borne illness in the Northern Hemisphere (1). LB is caused by spirochetes of the *Borrelia burgdorferi sensu lato* complex (Bbsl), which are transmitted to humans by Ixodidae ticks (2). The incidence of LB is rising, due to expanding tick populations, climate change, and other drivers (3, 4). Four hundred seventy-six thousand new cases of LB are predicted per year in the United States of America and 128,000–200,000 in Western Europe, which is likely an underestimation (5–7). Meta-analysis studies estimate 3.9%–13.6% Bbsl seroprevalence in Europe (8), with as high as 14.5% globally, albeit more studies are needed to improve the accuracy of global LB burden estimates (9).

LB symptoms often overlap with other conditions, making accurate and timely diagnosis a considerable challenge. Early clinical manifestations of LB may include non-specific systemic symptoms such as malaise, fatigue, myalgia, and headaches. A common early manifestation is the localized skin lesion erythema migrans (EM) (10). However, the infection can disseminate and within weeks involve the peripheral and central nervous system causing Lyme neuroborreliosis (LNB) or occasionally the heart, causing Lyme carditis. Late manifestations, which occur months to years after initial infection, include Lyme arthritis, where usually one or more of the larger joints are affected, and acrodermatitis chronica atrophicans (ACA), a chronic skin condition (11, 12). Early diagnosis and treatment are essential to prevent further disease progression and reduce persistent symptoms.

LB is caused by multiple Bbsl species: *Borrelia burgdorferi sensu stricto* (*ss*), *Borrelia afzelii*, and *Borrelia garinii*, and less commonly *Borrelia mayonii*, *Borrelia bavariensis*, and *Borrelia spielmanii*. In Europe, LB is primarily caused by *B. afzelii* or *B. garinii*, with a smaller contribution from *B. burgdorferi ss*, *B. bavariensis*, and *B. spielmanii* (1). *B. afzelii* more commonly results in skin manifestations, and it is almost exclusively associated with ACA, while *B. garinii* is linked to classic LNB (13, 14). In Asia, *B. garinii* is the leading causative species of LB (5, 15). In North America, most of the LB cases are attributed to *B. burgdorferi ss*, while a small number of cases are associated with the recently identified *B. mayonii* species (16, 17). In the Northeastern and mid-Atlantic United States, *B. burgdorferi ss* is notably arthritogenic, leading to a higher prevalence of Lyme arthritis cases in North America compared with Europe or Asia (1, 5). Importantly, these regional variations in species distribution can potentially impact the performance of laboratory diagnostics (18, 19).

LB, manifesting as EM, can be clinically diagnosed without the aid of laboratory tests. Clinical guidelines from the United States and Europe (20–22) currently recommend against the use of tests for diagnosing LB during the EM phase, partly because of their sub-optimal performance and an early window of the so-called serological silence, where antibody levels are not yet detectable. However, 20%–30% of infected cases may not develop EM (23, 24); thus, laboratory tests are necessary to support the diagnosis of LB. Direct detection methods such as PCR and tissue culture are not used in routine clinical diagnostics due to low sensitivity, stemming from the inconsistent presence of *B. burgdorferi* components in clinical samples (25). Serology is the primary laboratory method of LB detection due to its widespread availability, ease of use, and capacity for adaptation and improvement. Currently, a two-tiered serodiagnostic approach is recommended for the diagnosis of LB, which includes enzyme immunoassays (EIAs) and/or immunoblotting (26–28). These tests rely on either whole-cell lysates (WCLs) of Bbsl; recombinant antigens (proteins/peptides) from the most immunogenic Bbsl proteins such as the Vmp-like sequence, expressed (VlsE), outer surface proteins (Osps), or flagellin; or a combination of WCLs and recombinant antigens (25, 29). WCLs and recombinants used in the LB tests may be derived from a single genospecies, but given the marked Bbsl species diversity, this may impact the effectiveness of these assays. While the current tests serve as valuable diagnostic tools especially in the late stages of LB, they suffer from limitations leading to suboptimal sensitivity during the early stages of infection. Moreover, the immunoblot step is liable to variability in interpretation and false positive results may lead to overdiagnosis. Sensitivity of two-tiered testing remains low (30%–40%) during early infection, increasing to 70%–100% for disseminated and late-presenting LB (27). Although two-tiered testing demonstrates high specificity (>95%), false-positive outcomes still arise due to cross-reactivity with antigens from closely related microorganisms (27). Furthermore, the existing tests lack the capability to differentiate between current and past infection. The limitations of the current tests stem from their inability to fully capture the antigenic complexity of the *Borrelia* species and the host’s heterogenous immune response. The inclusion of more antigens expressed in the different stages of LB infection from different Bbsl genospecies in one test would overcome some of these persistent challenges.

This study investigates the performance of a novel enzyme-linked immunosorbent assay (ELISA), which uses a composite of multi-epitope chimeric antigens fused and displayed on a stable non-reactive protein scaffold to facilitate expression, correct folding, and orientation of the relevant peptides. The peptides utilized are heterogenous in size covering short peptides to subunits in order to capture antibodies to both linear and conformational epitopes. The study compares the diagnostic performance of the multi-epitope Epitogen Lyme Detect IgG ELISA with the LIAISON Borrelia IgG CLIA.

## MATERIALS AND METHODS

### Epitogen Lyme Detect IgG Development and design

**Fig 1:**
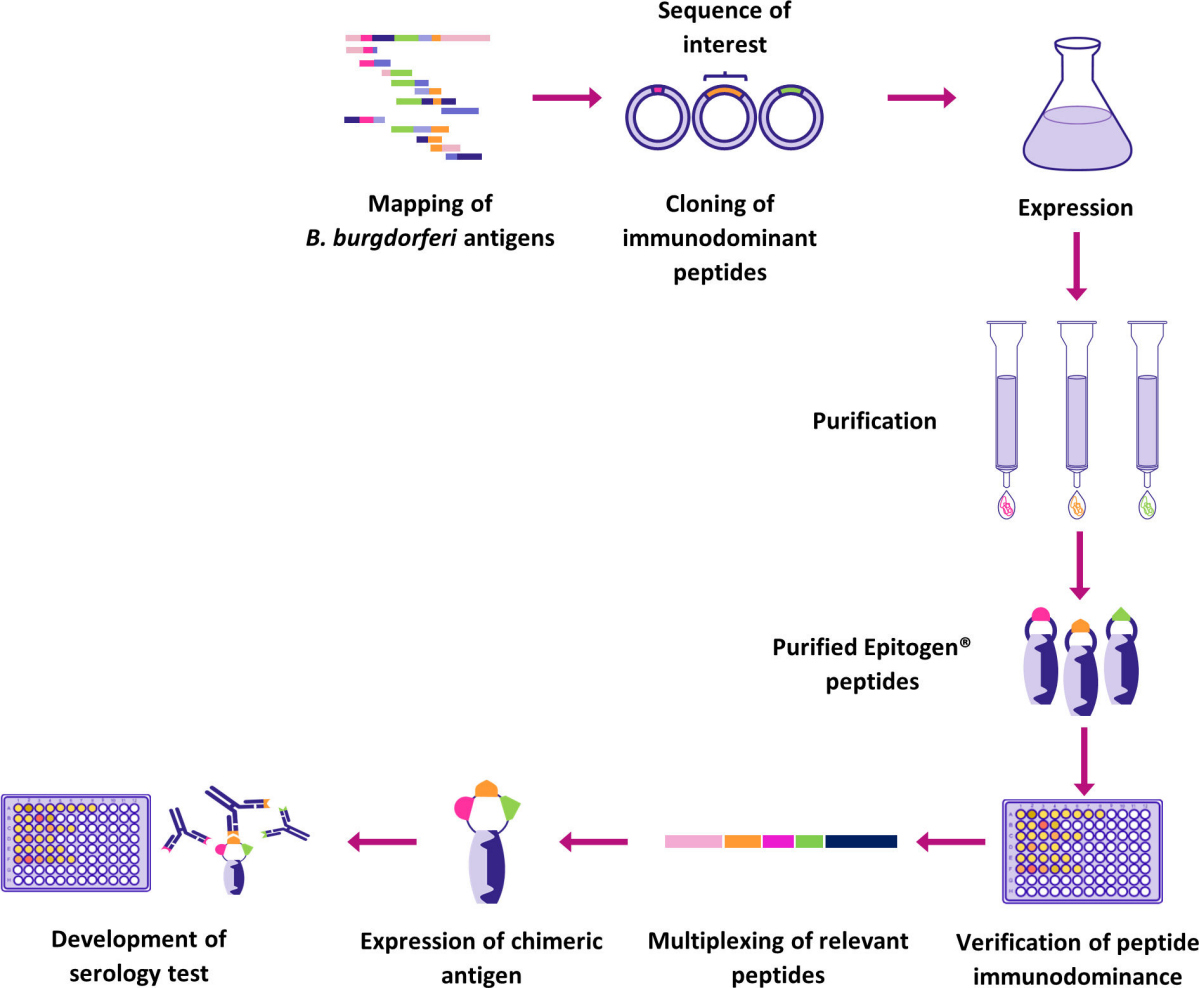
Epitogen Lyme Detect IgG Assay Development. Over 500 potential immunodominant Bbsl epitopes were first identified through *in silico* prediction tools and existing literature. The selected peptides were compared bioinformatically using the sequences from the four major pathogenic species. Variable peptides from different species were included in the final peptide selection. The DNA corresponding to each epitope was synthesized and then individually cloned into the Epitogen plasmid construct, expressed in *E. coli*, followed by cell lysis and purification. Once purified, each peptide was evaluated for immunodominance and cross-reactivity using 175 Lyme-positive and 125 Lyme-negative sera (data not shown). One hundred twenty seroreactive peptides with limited cross-reactivity were selected from 37 antigenic proteins.

The Epitogen Lyme Detect IgG assay is an indirect ELISA used to qualitatively detect IgG antibodies against Bbsl peptides in human serum or plasma (Fig. 1). Multi-epitope antigens were created by fusing 120 immunodominant and specific epitopes selected from 37 antigenic proteins of the major pathogenic species of Bbsl (Table 1). Five to eight peptides were fused together with the Epitogen scaffold forming each multi-peptide antigen, which served as the foundation for the test. The relevant epitopes displayed on one scaffold were separated by a flexible GS linker (GGGSGGG). This linker prevents interference from each other and provides freedom of individual peptide to rotate and form a stable conformation. The DNA encoding the relevant polyepitopes were codon optimized for the expression host (*Escherichia coli*) and synthesized commercially at GenScript. The DNA fragment was then cloned into the scaffold construct using a NEBuilder HiFi DNA Assembly Cloning Kit. The expression of the multi-peptide chimeric antigen from the construct, purification, and refolding of the purified protein were performed using standard methods (30, 31). The protein concentration was quantified on SDS-PAGE gel using bovine serum albumin as a reference. The ELISA microplate was coated with 200 ng per well of Bbsl multi-epitope antigen scaffolds. The chimeric antigens that demonstrated cross-reactivity (i.e., immunodominant hotspots derived from flagellin) were used at the lower concentration levels (50 ng/mL), while those antigens that demonstrated high specificity were used at a higher concentration of 200 ng/mL.

**TABLE 1:** The 37 antigenic targets along with their genomic loci in the *B. burgdorferi* reference strain B31^*a*^

Protein	Gene locus	Protein	Gene locus	Protein	Gene locus
APA	BB0366	Enolase	BB0337	Lmp1	BB0210
BdrA	BBP34	ErpG	BBS41	NAP, P22	BB0365
BdrP	BBL27	ErpL	BBO39	OppA2	BB0329
BdrQ	BBN34	ErpO/B1	BBL40	OppAIV	BBB16
BdrT	BBG33	ErpP (CRASP-3)	BBN38	OspA	BBA15
BdrU	BBH13	ErpQ	BBN39	OspB	BBA16
BdrV	BBQ42	FBP	BBK32	OspC	BBB19
BdrW	BBQ34	FlaA	BB0668	P35	BBI36
BmpA	BB0832	FlaB, P41	BB0147	P45	BBA57
CRASP1	BBA68	FlgE	BB0283	P66	BB0603
CRASP2	BBH06	FliL	BB0279	P83/100	BB0744
DBPA	BBA24	Lipoprotein	BBK07	VlsE	BBF0041
DBPB	BBA25				

^*a*^ These candidates were shown expressed in the literature and tested for antibody reactivity during assay development.

**Fig 2:**
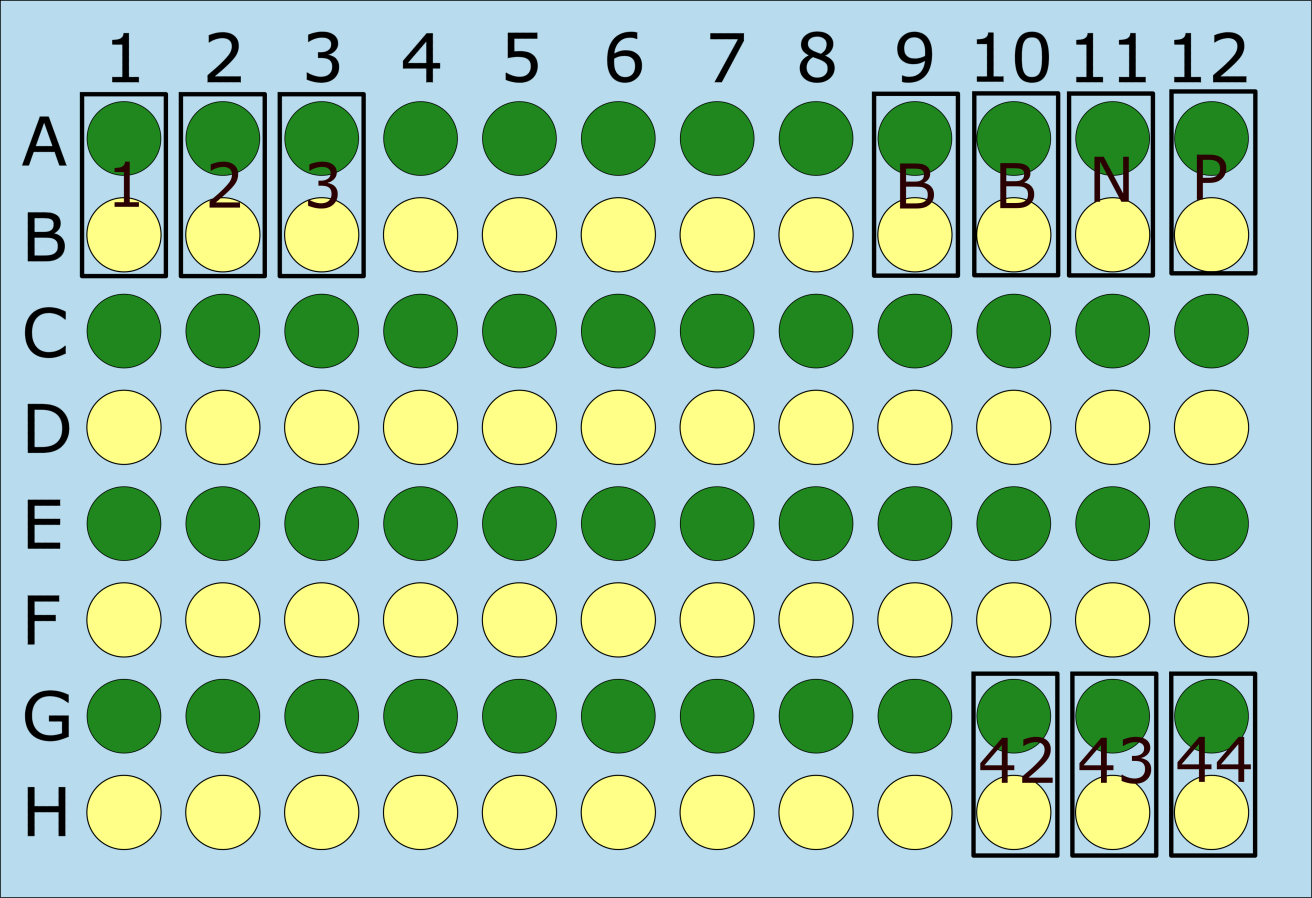
Epitogen Lyme Detect IgG Assay layout and sample distribution. Antigen coating: (green)—a set of composite IgG immunodominant peptides comprising 120 peptides from 37 Borrelia antigens; (yellow)—the Epitogen scaffold protein (control). Sample distribution: test sample (1 – 44)—44 single tests; blank (B)—sample diluent buffer; negative (N)—Lyme disease-negative serum sample; positive (P)—Lyme disease-positive serum sample.

Half of the total number of wells in a microwell plate is coated with the 120 immune dominant peptides multiplexed on a non-reactive Epitogen scaffold protein (Fig. 2). The other half of the wells in the microwell plate are coated with the Epitogen scaffold alone.

### Study design

A panel of 220 LB patient sera (Table 2) were tested using LIAISON Borrelia IgG CLIA (Diasorin, Saluggia, Italy) and Epitogen Lyme Detect IgG ELISA (EpitogenX, Aberdeen, UK). The samples were tested in a non-blinded fashion at the Scottish Lyme Disease and Tick-borne Infections Reference Laboratory (SLDTRL, Inverness, UK), following the manufacturer’s instructions and employing designated cut-off criteria. A further panel of 198 serum/plasma samples (Table 3), including samples from individuals with problematic infections/diseases known to cross-react with LB serology tests as well as healthy individuals, was non-blind tested using Epitogen Lyme Detect IgG assay (EpitogenX, Aberdeen, UK). The study samples were tested once to mimic testing in the routine clinical setting. Assay reproducibility was assessed using a positive and a negative control in 11 separate microwell plates.

**TABLE 2:** LB sample characteristics^*a*^

LB clinical manifestation	Samples per group number (% of total)
LNB	30 (13.6)
ACA	6 (2.7)
Lyme arthritis	24 (10.9)
EM	160 (72.7)
Acute (<6 weeks)	79 (35.9)
Early convalescent (6–12 weeks)	52 (23.6)
Late convalescent (>12 weeks)	29 (13.2)
Total	220 (100)

^*a*^ Acrodermatitis chronica atrophicans (ACA).

**TABLE 3:** Control sample characteristics

Group	Samples per group number (% of total)
Cross-reactivity controls	98 (49.5)
Cytomegalovirus	20 (10.1)
Epstein-Barr virus	20 (10.1)
Syphilis	30 (15.2)
Rheumatoid arthritis	17 (8.6)
Multiple sclerosis	11 (5.6)
Healthy individuals	100 (50.5)
Total	198 (100)

### Epitogen Lyme Detect IgG assay procedure (indirect ELISA)

The Epitogen Lyme Detect IgG plate was washed once with wash buffer (PBS + 0.05% Tween 20). Serum samples, positive and negative controls (EpitogenX, Aberdeen, UK), were diluted 1:101 in sample dilution buffer. One hundred microliters of each diluted sample/control as well as sample diluent buffer was added to two separate wells, one containing the antigen multiplex-Epitogen scaffold and the second, inert Epitogen scaffold only (Fig. 2). The microwell plate was incubated for 60 min at room temperature (RT). The plate was washed five times with washing buffer. One hundred microliters of anti-human IgG-HRP conjugate (1:10,000 in diluent buffer) was added to each well, and the plate was incubated for 30 min at RT. The plate was washed five times with washing buffer. One hundred microliters of 3,3′,5,5′-Tetramethylbenzidine was added to each well. The plate was incubated for 5 min at RT, and the reaction stopped with 100 µL 2M H_2_SO_4_. The optical density (OD) was read at λ = 450 nm by a spectrophotometer.

The ODs from both antigen and antigen control wells together with the ODs from both sample diluent control (blank) wells were used for assay index calculations and result interpretation. Index was calculated by antigen-specific OD divided by the mean OD of the sample diluent control (blank). The antigen-specific OD is the OD from the well coated with antigens on the Epitogen scaffold (Ag) subtracted by the OD from the paired control well coated with the Epitogen scaffold only (Ag Ctl).


$${Index}_{Sample\;A}=\frac{{OD}_{Ag(Sample\;A)}-{OD}_{Ag\;Ctl(Sample\;A)}}{{\bar{x}}_{OD(blank)}}$$

An Index < 2.5 was considered negative, whereas an Index ≥ 2.5 was reactive.

### Assay sensitivity

Epitogen Lyme Detect IgG sensitivity was determined by retrospectively testing stored sera selected from samples, which had been referred to the SLDTRL for laboratory diagnosis of LB between 2018 and 2023. All samples had previously been tested by standard two-tier testing: LIAISON Borrelia IgG and LIAISON Borrelia IgM Quant/II CLIA on the Liaison XL analyzer (Diasorin) followed by Borrelia recomLine IgG and IgM immunoblot (Mikrogen, Germany) on the CarL immunoblot platform (Mikrogen) if reactive (positive/equivocal). In total, 220 sera were tested, including 160 from patients with suspected EM, 30 from patients with confirmed LNB (as per European Federation of Neurological Societies guidelines), 6 from patients with ACA, and 24 from patients with Lyme arthritis (Table 2). Suspected EM patients were categorized based on the date of serum collection after onset of EM symptoms/date of exposure: acute < 6 weeks (but more than 1 week), early convalescent 6–12 weeks, and late convalescent > 12 weeks (but less than 1 year). Epitogen Lyme Detect IgG results were compared with those obtained with the LIAISON Borrelia IgG.

### Assay specificity

Epitogen Lyme Detect IgG specificity was determined by testing 100 normal serum samples denoted as healthy individuals and 96 sera as well as 2 plasma samples from individuals with diseases that are humorally similar (Table 3). All control samples were purchased from Precision for Medicine (Norton, MA, US). Healthy individuals (*n* = 100) were tested negative for human immunodeficiency viruses, Hepatitis B surface antigen, and Hepatitis C by EIAs or Procleix Ultrio Elite Assay Procleix Panther system (Grifols Diagnostic Solutions Inc., Emeryville, CA, US). The problematic sample groups contained individuals confirmed positive for cytomegalovirus (CMV, *n* = 20) and EBV (*n* = 20) infections, as well as clinically diagnosed cases of syphilis (*n* = 30), rheumatoid arthritis (RA, *n* = 17), and multiple sclerosis (MS, *n* = 11). All the control samples were assumed LB negative, albeit none of the control samples were tested for LB.

### Statistical analysis

The 95% confidence intervals (CIs) of the sensitivity and specificity were calculated using the method of Clopper and Pearson. Exact McNemar test was used to compare assay results from the Epitogen Lyme Detect IgG with the LIAISON Borrelia IgG. Fisher’s exact test was employed to compare the diagnostic outcome of two assays that used a different set of samples. For all analyses, *P*  <  0.05 was considered statistically significant.

## RESULTS

Coefficient of variation values of the Epitogen Lyme Detect IgG assay were below 10%. The average Index of the positive control sample was 17.83 (SD = 1.06, CV = 5.97%). The average OD of blank readout was 0.045 (SD = 0.004, CV = 9.14%); the average OD of the negative control sample was 0.148 (SD = 0.005, CV = 3.35%); the average OD of the positive control sample was 0.844 (SD = 0.037, CV = 4.39%).

Both LIAISON Borrelia IgG and Epitogen Lyme Detect IgG demonstrated a sensitivity of 100% with LNB, ACA, and Lyme arthritis patients (Table 4). However, the Epitogen Lyme Detect IgG showed a significantly higher sensitivity with suspected EM patients compared with the LIAISON Borrelia IgG (67.5% and 54.4% respectively; *P* < 0.001, *n* = 160). Acute and early convalescent EM groups had the lowest sensitivity, 53.2% and 44.2%, respectively, with the LIAISON Borrelia IgG, but this was significantly higher (65.8% and 63.5%, respectively; *P* < 0.01) with the Epitogen Lyme Detect IgG. Both assays showed increased sensitivity in detecting late convalescent EM (75.9% with LIAISON Borrelia IgG, 79.3% with Epitogen Lyme Detect IgG). Considering all of the LB manifestations used in the study, the Epitogen Lyme Detect IgG exhibited a significantly higher sensitivity (76.4%) compared with the LIAISON Borrelia IgG (66.8%; *P* < 0.001, *n* = 220). 1.8% (4 out of 220) of the reactive LIAISON samples was equivocal. Epitogen Lyme Detect IgG successfully detected all samples that were reactive (positive or equivocal) with the LIAISON Borrelia IgG.

**TABLE 4:** Sensitivity of LIAISON Borrelia IgG and Epitogen Lyme Detect IgG assays^*a*^^,^^*b*^

	No. reactive [sensitivity (%); 95% CI]		
	LIAISON	LIAISON	Epitogen
	Positive and equivocal	Positive	Positive
LNB (*n* = 30)	30 (100; 88.4–100)	30 (100; 88.4–100)	30 (100; 88.4–100)
ACA (*n* = 6)	6 (100; 54.1–100)	6 (100; 54.1–100)	6 (100; 54.1–100)
Lyme arthritis (*n* = 24)	24 (100; 85.8–100)	23 (95.8; 78.9–99.9)	24 (100; 85.8–100)
EM (*n* = 160)	87^*e*^ (54.4; 46.3–62.3)	84^*f*^ (52.5; 44.5–60.4)	108^*e*^^,^^*f*^ (67.5; 59.7–74.7)
Acute <6 weeks (*n* = 79)	42^*c*^ (53.2; 41.6–64.5)	39^*f*^ (49.4; 37.9–60.9)	52^*c*^^,^^*f*^ (65.8; 54.3–76.1)
Early convalescent 6–12 weeks (*n* = 52)	23^*c*^ (44.2; 30.5–58.7)	23^*d*^ (44.2; 30.5–58.7)	33^*c*^^,^^*d*^ (63.5; 49.0–76.4)
Late convalescent >12 weeks (*n* = 29)	22 (75.9; 56.5–89.7)	22 (75.9; 56.5–89.7)	23 (79.3; 60.3–92.0)
Total (*n* = 220)	147^*e*^ (66.8; 60.2–73.0)	143^*f*^ (65.0; 58.3–71.3)	168^*e*^^,^^*f*^ (76.4; 70.2–81.8)

^*a*^ The reactivity analysis of the LIAISON Borrelia IgG test was divided into two distinct groups, with the first group defining positive and equivocal results as reactive, whereas the second group considered only positive results as reactive. Solely positive Epitogen Lyme Detect IgG assay result was considered reactive.

^*b*^ Acrodermatitis chronica atrophicans (ACA).

^*c*^ *P* < 0.01 comparing Epitogen Lyme Detect IgG (positive) and LIAISON Borrelia IgG (positive and borderline).

^*d*^ *P* < 0.01 comparing Epitogen Lyme Detect IgG (positive) and LIAISON Borrelia IgG (positive).

^*e*^ *P* < 0.001 comparing Epitogen Lyme Detect IgG (positive) and LIAISON Borrelia IgG (positive and borderline).

^*f*^ *P* < 0.001 comparing Epitogen Lyme Detect IgG (positive) and LIAISON Borrelia IgG (positive).

Specificity of the Epitogen Lyme Detect IgG assay was evaluated using 198 samples. Epitogen Lyme Detect IgG showed no cross-reactivity with samples from patients with CMV (0 out of 20) and MS (0 out of 11) (Table 5). There was 5% cross-reactivity with samples from patients with EBV (1 out of 20) and 5.9% with RA (1 out of 17). The highest false-positive rate of 10% was observed in the Syphilis group (3 out of 30). The cross-reactivity was 4.0% in the group of healthy individuals (4 out of 100). Based on 198 samples used in this study, the specificity of the Epitogen Lyme Detect IgG was 95.5% (95% CI 91.6–97.9) (Table 5).

**TABLE 5:** Specificity of Epitogen Lyme Detect IgG assay

	No. cross-reactive [cross-reactivity (%); 95% CI]	No. non-reactive [specificity (%); 95% CI]
	Epitogen Lyme Detect IgG	Epitogen Lyme Detect IgG
Cross-reactivity controls (*n* = 98)	5	93
	(5.1; 1.7–11.5)	(96.9; 91.3–99.4)
CMV (*n* = 20)	0 (0; 0–16.8)	20 (100; 83.2–100)
EBV (*n* = 20)	1 (5.0; 0–24.9)	19 (95.0; 75.1–99.9)
Syphilis (*n* = 30)	3 (10; 2.1–26.5)	27 (90.0; 73.5–97.9)
RA (*n* = 17)	1 (5.9; 0–28.7)	16 (94.1; 71.3–99.9)
MS (*n* = 11)	0 (0; 0–28.5)	11 (100; 71.5–100)
Healthy controls (*n* = 100)	4 (4.0; 1.1–9.9)	96 (96.0; 90.1–98.9)
Total (*n* = 198)	9 (4.6; 2.1–8.5)	189 (95.5; 91.6–97.9)

The overall specificity of the Epitogen Lyme Detect IgG was then compared with the specificity of the LIAISON Borrelia IgG from previously published studies (29, 32, 33) using Fisher’s exact test (Table 6). No significant differences were found.

**TABLE 6:** Comparison of published LIAISON Borrelia IgG specificity studies with the specificity of the Epitogen Lyme Detect IgG

Test	No. non-reactive/healthy Individuals [specificity (%); 95% CI]	No. non-reactive/total [specificity (%); 95% CI]	Study
Epitogen	96/100 (96.0; 90.1–98.9)	189/198 (95.5^*a*^; 91.6–97.9)	Current study
LIAISON	219/234 (93.6; 89.7–96.4)	255/274 (93.1^*a*^; 89.4–95.8)	Marangoni et al. (33)
LIAISON	48/50 (96.0; 86.3–99.5)	138/151 (91.4^*a*^; 85.7–95.3)	Busson et al. (32)
LIAISON	71/74 (95.9; 90.5–99.7)	112/122 (91.8^*a*^, 85.4–96.0)	Hoeve-Bakker et al. (29)

^*a*^ *P* > 0.05 (no statistical difference) comparing Epitogen Lyme Detect IgG with three separate LIAISON Borrelia IgG test studies using Fisher’s exact test.

## DISCUSSION

This study provides a valuable insight into the diagnostic performance of the novel multiple epitope-based Epitogen Lyme Detect IgG assay. In summary, the Epitogen Lyme Detect IgG ELISA was as sensitive as the LIAISON Borrelia IgG CLIA in patients with LNB, ACA, and Lyme arthritis. However, the Epitogen Lyme Detect IgG significantly outperformed the LIAISON Borrelia IgG in acute (<6 weeks), early convalescent (6–12 week), and late convalescent (>12 weeks) suspected EM, and consequently, the overall sensitivity was higher. The specificity of the Epitogen Lyme Detect IgG was high and comparable with the reported specificity of the LIAISON Borrelia IgG.

The diagnosis of LB can be challenging. The development of detectable antibody levels in response to infection with Bbsl can vary from several days to a few weeks depending on the individual’s immune system, the infecting genospecies, and other factors (25, 34). Furthermore, patients with early LB may remain seronegative if treated promptly with antibiotics (27, 35). Detection of IgM antibodies can be used for the laboratory diagnosis of early LB of up to 6 weeks (36). However, the benefits and limitations of IgM serology tests for early LB detection vary across different regions in Europe, leading to a lack of consensus regarding their use (37, 38). The presence of EM is clinically diagnostic of early LB. Patients should be treated empirically, and serology testing is not recommended due to the potential for false-negative results (12). However, only 70%–80% of patients with LB develop EM and not all rashes are classic, leading to clinical uncertainty and underscoring the necessity for accurate testing during the early stages of the illness. When evaluating laboratory results, it is important to consider the limitations of current tests, including the choice and the number of antigenic proteins used.

Even after nearly three decades and despite the documented deficiencies, the two-tiered testing strategy remains the accepted method for the laboratory diagnosis of LB (27, 39). Immunoblots are used as a second-stage confirmatory test due to the ability to distinguish antibodies to individual proteins providing greater specificity (40) and useful additional information about the duration of infection (41). However, the increased specificity comes at the cost of lower sensitivity in early LB diagnosis (41, 42) and immunoblots can be prone to subjective interpretation (43). Densitometric blot analysis aids in determining whether bands are too faint to be scored (44). However, the complexity of immunoblot protocols and interpretation prevent many clinical laboratories from offering the test on-site. The sequential use of two different EIAs instead of the immunoblot step offers a result that is less complex to interpret, less expensive to run, and without the need for special expertise (45). Conversely, removing immunoblotting may result in a loss of valuable clinical data about the extent and the maturity of the antibody response, therefore impacting the ability to determine the stage of infection (36). While serology tests have advanced, existing assays remain a long way from accurately detecting early LB cases and distinguishing between current infection and past exposure. These limitations underscore the need for both improved direct testing and better serology methods. A promising avenue to address the shortcomings of LB diagnosis involves further research into epitope-based serology approaches, multiplexing a list of epitopes associated with early diagnosis of LB and/or differentiation between active and past infections. Indeed, studies utilizing the immunodominant regions approach show improved accuracy in early LB (46, 47), suggesting that peptide-based serology assays may be valuable for detecting early LB.

The choice of antigens impacts the sensitivity and specificity of EIAs. Traditionally, assays using WCLs were utilized for the laboratory diagnosis of LB, which could theoretically detect multiple Bbsl genospecies (48). However, WCL-based tests lacked crucial *in vivo*-expressed antigens such as VlsE (49). They contained a significant proportion of non-immunogenic epitopes, leading to a reduced sensitivity. Additionally, many WCL proteins are homologous among unrelated microorganisms inevitably leading to cross-reactivity and false positives (29). Newer generation assays employ a selection of a few recombinant proteins or synthetic peptides, such as VlsE, OspC, PepC10, and others (50). Studies have shown that EIAs utilizing a limited number of antigens demonstrate increased specificity and sensitivity compared with WCLs (26, 29). However, the high sequence variability of antigens between the various Bbsl genospecies could potentially impact the performance of these assays unless a collection of antigens from the different genospecies are included. Also, the heterogenous nature of the population immune system means that there may be no or limited antibody response to these recombinant antigens and synthetic peptides, which in turn will impact assay sensitivity. The LIAISON Borrelia IgG CLIA utilizes the VlsE antigen from *B. garinii*, which is expected to have high sensitivity as it contains highly conserved regions between the different Bbsl genospecies. Conversely, the Epitogen Lyme Detect IgG assay incorporates a wide range of Bbsl antigens from multiple genospecies, possibly explaining the observed increase in sensitivity and thus showing its potential suitability to cover more geographic areas.

Recent LB test developments have shifted toward peptide-based serology approaches demonstrating improved sensitivity and specificity. First, given the antigenic variability among the Bbsl genospecies, their considerable genome sizes, and the tendency of these bacteria to alter protein expression during different infection stages, incorporating a diverse range of Bbsl antigens into the assay is crucial for enhancing the test performance. Studies have shown that enhanced test sensitivity correlates with the number of antigens used, especially in the early stages of LB when the immune response may be more varied or less pronounced (46, 47). Second, including multiple immunodominant epitopes, which cover the most prevalent pathogenic Bbsl genospecies, improves assay sensitivity. This approach also allows the elimination of potential cross-reactive epitopes, thereby enhancing assay specificity (51, 52). Furthermore, using multiple antigen/peptide panels may help distinguish between different clinical stages of the disease (53). The multi-epitope strategy has previously been employed in developing point-of-care serodiagnostic tests for LB (54, 55), though focusing only on short linear peptides may impact overall sensitivity. In line with expectations for peptide-based assays, the Epitogen Lyme Detect IgG demonstrates improved sensitivity and high specificity. Both healthy and cross-reactivity control groups showed similar specificity (96.0% vs. 96.9%) in the Epitogen Lyme IgG assay, whereas the cross-reactivity group often showed high cross-reaction in other Lyme IgG assays. It is noteworthy that none of the control samples were confirmed Bbsl-specific antibody negative or had no history of Bbsl exposure. Considering the 3.9%–13.6% Bbsl seroprevalence in Europe (8), the specificity figures of the Epitogen Lyme Detect IgG assay could be further improved by using Bbsl-specific antibody negative samples in future studies.

Multi-epitope-based assays, especially those that can capture both short linear and large conformational epitopes, have the potential to improve current diagnostic performance. Given the high accuracy, it is suggested that multiplexed serologic assays for LB have potential as standalone tests without a two-tiered approach (40), including testing of patients with early LB (56). Furthermore, assays designed utilizing a multiplexing approach demonstrate adaptability. For instance, in the event that the research community identifies new epitopes or emerging genospecies, those epitopes can be seamlessly integrated into the test, thereby enhancing its accuracy. Likewise, antigens used in vaccines can be excluded. Furthermore, epitope-based tests could be used to develop genospecies-specific testing for LB, providing valuable epidemiological insights, and might increase diagnostic accuracy, although testing may be more complex and costly compared with broad-spectrum tests.

It is worth noting that the study includes LB patients solely from Scotland, hence introducing selection bias. However, this study reflects “real-world” samples from clinical practice, which may involve uncertainties regarding the diagnosis of EM, the timing of exposure and/or EM, and the timing of antibiotic treatment relative to exposure. Subsequent studies should re-evaluate the Epitogen Lyme Detect IgG performance using a larger panel of well-characterized and suspected LB patient samples, including samples collected from longitudinal studies, as well as geographically matched samples and controls from Europe, North America, and Asia. This would capture the diverse pathogenic Bbsl distribution across different regions and reveal the universal suitability of the Epitogen Lyme Detect IgG assay to detect the various Bbsl species. Potentially cross-reactive groups could be further expanded, for example, including relapsing fever Borrelia. Further studies should test LB and control samples using multiple tests and technical repeats ensuring better comparison among assays.

In conclusion, the novel epitope-based Epitogen Lyme Detect IgG ELISA shows high and comparable accuracy in disseminated and late LB patient groups and healthy individuals, while having a superior sensitivity in suspected EM cases compared with the conventional test, indicating its potential for more accurate and timely diagnosis of LB. Likewise, the epitope approach can be employed to develop highly precise and specific IgM assays to LB further improving the early diagnosis of LB patients. Epitogen technology can readily integrate new antigens corresponding to emerging genospecies while excluding those used in vaccines, thereby offering a versatile solution to evolving diagnostic needs.

## References

[1] Stanek G, Wormser GP, Gray J, Strle F. 2012. Lyme borreliosis. Lancet 379:461–473. doi:10.1016/S0140-6736(11)60103-7 21903253

[2] Steere A.C. 2001. Lyme disease. N Engl J Med 345:115–125. doi:10.1056/NEJM200107123450207 11450660

[3] Brownstein JS, Holford TR, Fish D. 2005. Effect of climate change on Lyme disease risk in North America. Ecohealth 2:38–46. doi:10.1007/s10393-004-0139-x 19008966 PMC2582486

[4] Mead P. 2022. Epidemiology of Lyme disease. Infect Dis Clin North Am 36:495–521. doi:10.1016/j.idc.2022.03.004 36116831

[5] Steere AC, Strle F, Wormser GP, Hu LT, Branda JA, Hovius JWR, Li X, Mead PS. 2016. Lyme borreliosis. Nat Rev Dis Primers 2:16090. doi:10.1038/nrdp.2016.90 27976670 PMC5539539

[6] Kugeler KJ, Schwartz AM, Delorey MJ, Mead PS, Hinckley AF. 2021. Estimating the frequency of Lyme disease diagnoses, United States, 2010–2018. Emerg Infect Dis 27:616–619. doi:10.3201/eid2702.202731 33496229 PMC7853543

[7] Burn L, Tran TMP, Pilz A, Vyse A, Fletcher MA, Angulo FJ, Gessner BD, Moïsi JC, Jodar L, Stark JH. 2023. Incidence of Lyme borreliosis in Europe from national surveillance systems (2005–2020). Vect-Borne Zoon Dis 23:156–171. doi:10.1089/vbz.2022.0071 PMC10122223 37071405

[8] Burn L, Pilz A, Vyse A, Gutiérrez Rabá AV, Angulo FJ, Tran TMP, Fletcher MA, Gessner BD, Moïsi JC, Stark JH. 2023. Seroprevalence of Lyme borreliosis in Europe: results from a systematic literature review (2005–2020). Vect-Borne Zoon Dis 23:195–220. doi:10.1089/vbz.2022.0069 PMC10122246 37071401

[9] Dong Y, Zhou G, Cao W, Xu X, Zhang Y, Ji Z, Yang J, Chen J, Liu M, Fan Y, Kong J, Wen S, Li B, Yue P, Liu A, Bao F. 2022. Global seroprevalence and sociodemographic characteristics of Borrelia burgdorferi sensu lato in human populations: a systematic review and meta-analysis. BMJ Glob Health 7:e007744. doi:10.1136/bmjgh-2021-007744 PMC9185477 35697507

[10] Cardenas-de la Garza JA, De la Cruz-Valadez E, Ocampo-Candiani J, Welsh O. 2019. Clinical spectrum of Lyme disease. Eur J Clin Microbiol Infect Dis 38:201–208. doi:10.1007/s10096-018-3417-1 30456435

[11] Steere AC. 1989. Lyme disease. N Engl J Med 321:586–596. doi:10.1056/NEJM198908313210906 2668764

[12] Stanek G, Fingerle V, Hunfeld K-P, Jaulhac B, Kaiser R, Krause A, Kristoferitsch W, O’Connell S, Ornstein K, Strle F, Gray J. 2011. Lyme borreliosis: clinical case definitions for diagnosis and management in Europe. Clin Microbiol Infect 17:69–79. doi:10.1111/j.1469-0691.2010.03175.x 20132258

[13] Strle F, Stanek G. 2009. Clinical manifestations and diagnosis of Lyme borreliosis. Curr Probl Dermatol 37:51–110. doi:10.1159/000213070 19367097

[12] Stanek G, Reiter M. 2011. The expanding Lyme Borrelia complex--clinical significance of genomic species? Clin Microbiol Infect 17:487–493. doi:10.1111/j.1469-0691.2011.03492.x 21414082

[15] Wang G, van Dam AP, Schwartz I, Dankert J. 1999. Molecular typing of Borrelia burgdorferi sensu lato: taxonomic, epidemiological, and clinical implications. Clin Microbiol Rev 12:633–653. doi:10.1128/CMR.12.4.633 10515907 PMC88929

[16] Mead PS. 2015. Epidemiology of Lyme disease. Infect Dis Clin North Am 29:187–210. doi:10.1016/j.idc.2015.02.010 25999219

[17] Pritt BS, Mead PS, Johnson DKH, Neitzel DF, Respicio-Kingry LB, Davis JP, Schiffman E, Sloan LM, Schriefer ME, Replogle AJ, Paskewitz SM, Ray JA, Bjork J, Steward CR, Deedon A, Lee X, Kingry LC, Miller TK, Feist MA, Theel ES, Patel R, Irish CL, Petersen JM. 2016. Identification of a novel pathogenic Borrelia species causing Lyme borreliosis with unusually high spirochaetaemia: a descriptive study. Lancet Infect Dis 16:556–564. doi:10.1016/S1473-3099(15)00464-8 26856777 PMC4975683

[18] Branda JA, Strle F, Strle K, Sikand N, Ferraro MJ, Steere AC. 2013. Performance of United States serologic assays in the diagnosis of Lyme borreliosis acquired in Europe. Clin Infect Dis 57:333–340. doi:10.1093/cid/cit235 23592827 PMC8210822

[19] Marques AR, Strle F, Wormser GP. 2021. Comparison of Lyme disease in the United States and Europe. Emerg Infect Dis 27:2017–2024. doi:10.3201/eid2708.204763 34286689 PMC8314816

[20] European Centre for Disease Prevention and Control. 2016. A systematic literature review on the diagnostic accuracy of serological tests for Lyme borreliosis. ECDC, Stockholm. https://data.europa.eu/doi/10.2900/309479.

[21] Cruickshank M, O’Flynn N, Faust SN, Guideline C. 2018. Lyme disease: summary of NICE guidance. BMJ 361:k1261. doi:10.1136/bmj.k1261 29650513

[22] Lantos PM, Rumbaugh J, Bockenstedt LK, Falck-Ytter YT, Aguero-Rosenfeld ME, Auwaerter PG, Baldwin K, Bannuru RR, Belani KK, Bowie WR, et al.. 2021. Clinical Practice Guidelines by the Infectious Diseases Society of America (IDSA), American Academy of Neurology (AAN), and American College of Rheumatology (ACR): 2020 guidelines for the prevention, diagnosis and treatment of Lyme disease. Clin Infect Dis 72:e1–e48. doi:10.1093/cid/ciaa1215 33417672

[23] Milner RM, Mavin S, Ho-Yen DO. 2009. Lyme borreliosis in Scotland is different. J Infect 59:146–147. doi:10.1016/j.jinf.2009.05.007 19535148

[24] Schwartz AM, Hinckley AF, Mead PS, Hook SA, Kugeler KJ. 2017. Surveillance for Lyme disease - United States, 2008-2015. MMWR Surveill Summ 66:1–12. doi:10.15585/mmwr.ss6622a1 PMC5829628 29120995

[25] Branda JA, Steere AC. 2021. Laboratory diagnosis of Lyme borreliosis. Clin Microbiol Rev 34:e00018-19. doi:10.1128/CMR.00018-19 PMC7849240 33504503

[26] Molins CR, Delorey MJ, Sexton C, Schriefer ME. 2016. Lyme borreliosis serology: performance of several commonly used laboratory diagnostic tests and a large resource panel of well-characterized patient samples. J Clin Microbiol 54:2726–2734. doi:10.1128/JCM.00874-16 27558183 PMC5078550

[27] Moore A, Nelson C, Molins C, Mead P, Schriefer M. 2016. Current guidelines, common clinical pitfalls, and future directions for laboratory diagnosis of Lyme disease, United States. Emerg Infect Dis 22:1169–1177. doi:10.3201/eid2207.151694 27314832 PMC4918152

[28] Pegalajar-Jurado A, Schriefer ME, Welch RJ, Couturier MR, MacKenzie T, Clark RJ, Ashton LV, Delorey MJ, Molins CR. 2018. Evaluation of modified two-tiered testing algorithms for Lyme disease laboratory diagnosis using well-characterized serum samples. J Clin Microbiol 56. doi:10.1128/JCM.01943-17 PMC6062810 29743307

[29] Hoeve-Bakker BJA, Jonker M, Brandenburg AH, den Reijer PM, Stelma FF, van Dam AP, van Gorkom T, Kerkhof K, Thijsen SFT, Kremer K. 2022. The performance of nine commercial serological screening assays for the diagnosis of Lyme borreliosis: a multicenter modified two-gate design study. Microbiol Spectr 10:e0051022. doi:10.1128/spectrum.00510-22 35297658 PMC9045392

[30] Wang T, Diaz-Rosales P, Costa MM, Campbell S, Snow M, Collet B, Martin SAM, Secombes CJ. 2011. Functional characterization of a nonmammalian IL-21: rainbow trout Oncorhynchus mykiss IL-21 upregulates the expression of the Th cell signature cytokines IFN-γ, IL-10, and IL-22. J Immunol 186:708–721. doi:10.4049/jimmunol.1001203 21160047

[31] Wang T, Hu Y, Wangkahart E, Liu F, Wang A, Zahran E, Maisey KR, Liu M, Xu Q, Imarai M, Secombes CJ. 2018. Interleukin (IL)-2 is a key regulator of T helper 1 and T helper 2 cytokine expression in fish: functional characterization of two divergent IL2 paralogs in salmonids. Front Immunol 9:1683. doi:10.3389/fimmu.2018.01683 30093902 PMC6070626

[32] Busson L, Reynders M, Van den Wijngaert S, Dahma H, Decolvenaer M, Vasseur L, Vandenberg O. 2012. Evaluation of commercial screening tests and blot assays for the diagnosis of Lyme borreliosis. Diagn Microbiol Infect Dis 73:246–251. doi:10.1016/j.diagmicrobio.2012.04.001 22560168

[33] Marangoni A, Sambri V, Accardo S, Cavrini F, Mondardini V, Moroni A, Storni E, Cevenini R. 2006. A decrease in the immunoglobulin G antibody response against the VlsE protein of Borrelia burgdorferi sensu lato correlates with the resolution of clinical signs in antibiotic-treated patients with early Lyme disease. Clin Vaccine Immunol 13:525–529. doi:10.1128/CVI.13.4.525-529.2006 16603623 PMC1459630

[34] Talagrand-Reboul E, Raffetin A, Zachary P, Jaulhac B, Eldin C. 2020. Immunoserological diagnosis of human borrelioses: current knowledge and perspectives. Front Cell Infect Microbiol 10:241. doi:10.3389/fcimb.2020.00241 32509603 PMC7248299

[35] Rebman AW, Crowder LA, Kirkpatrick A, Aucott JN. 2015. Characteristics of seroconversion and implications for diagnosis of post-treatment Lyme disease syndrome: acute and convalescent serology among a prospective cohort of early Lyme disease patients. Clin Rheumatol 34:585–589. doi:10.1007/s10067-014-2706-z 24924604

[36] Dessau RB, van Dam AP, Fingerle V, Gray J, Hovius JW, Hunfeld K-P, Jaulhac B, Kahl O, Kristoferitsch W, Lindgren P-E, Markowicz M, Mavin S, Ornstein K, Rupprecht T, Stanek G, Strle F. 2018. To test or not to test? Laboratory support for the diagnosis of Lyme borreliosis: a position paper of ESGBOR, the ESCMID study group for Lyme borreliosis. Clin Microbiol Infect 24:118–124. doi:10.1016/j.cmi.2017.08.025 28887186

[37] Hillerdal H, Henningsson AJ. 2021. Serodiagnosis of Lyme borreliosis-is IgM in serum more harmful than helpful? Eur J Clin Microbiol Infect Dis 40:1161–1168. doi:10.1007/s10096-020-04093-2 33409833 PMC8139919

[38] Joyner G, Mavin S, Milner R, Lim C. 2022. Introduction of IgM testing for the diagnosis of acute Lyme borreliosis: a study of the benefits, limitations and costs. Eur J Clin Microbiol Infect Dis 41:671–675. doi:10.1007/s10096-021-04366-4 35089441 PMC8934319

[39] Recommendations for test-performance and interpretation from the 2nd national conference on serologic diagnosis of Lyme-disease (Reprinted from MMWR, vol 44, pg 590-591, 1995). 1995. JAMA 274:937. doi:10.1001/jama.1995.03530120023018 7623762

[40] Branda JA, Body BA, Boyle J, Branson BM, Dattwyler RJ, Fikrig E, Gerald NJ, Gomes-Solecki M, Kintrup M, Ledizet M, et al.. 2018. Advances in serodiagnostic testing for Lyme disease are at hand. Clin Infect Dis 66:1133–1139. doi:10.1093/cid/cix943 29228208 PMC6019075

[41] Steere AC, McHugh G, Damle N, Sikand VK. 2008. Prospective study of serologic tests for lyme disease. Clin Infect Dis 47:188–195. doi:10.1086/589242 18532885 PMC5538270

[42] Wormser Gary P, Schriefer M, Aguero-Rosenfeld ME, Levin A, Steere AC, Nadelman RB, Nowakowski J, Marques A, Johnson BJB, Dumler JS. 2013. Single-tier testing with the C6 peptide ELISA kit compared with two-tier testing for Lyme disease. Diagn Microbiol Infect Dis 75:9–15. doi:10.1016/j.diagmicrobio.2012.09.003 23062467 PMC4052829

[43] Seriburi V, Ndukwe N, Chang Z, Cox ME, Wormser GP. 2012. High frequency of false positive IgM immunoblots for Borrelia burgdorferi in clinical practice. Clin Microbiol Infect 18:1236–1240. doi:10.1111/j.1469-0691.2011.03749.x 22369185

[44] Binnicker MJ, Jespersen DJ, Harring JA, Rollins LO, Bryant SC, Beito EM. 2008. Evaluation of two commercial systems for automated processing, reading, and interpretation of Lyme borreliosis Western blots. J Clin Microbiol 46:2216–2221. doi:10.1128/JCM.00200-08 18463211 PMC2446909

[45] Wormser G.P., Levin A, Soman S, Adenikinju O, Longo MV, Branda JA. 2013. Comparative cost-effectiveness of two-tiered testing strategies for serodiagnosis of Lyme disease with noncutaneous manifestations. J Clin Microbiol 51:4045–4049. doi:10.1128/JCM.01853-13 24068010 PMC3838032

[46] Brandt KS, Ullmann AJ, Molins CR, Horiuchi K, Biggerstaff BJ, Gilmore RD. 2019. Evaluation of in vivo expressed Borrelia burgdorferi antigens for improved IgM serodiagnosis of early Lyme disease. Diagn Microbiol Infect Dis 93:196–202. doi:10.1016/j.diagmicrobio.2018.09.012 30344068

[47] Reifert J, Kamath K, Bozekowski J, Lis E, Horn EJ, Granger D, Theel ES, Shon J, Sawyer JR, Daugherty PS. 2021. Serum epitope repertoire analysis enables early detection of Lyme disease with improved sensitivity in an expandable multiplex format. J Clin Microbiol 59. doi:10.1128/JCM.01836-20 PMC8111119 33148704

[48] Smismans A, Goossens VJ, Nulens E, Bruggeman CA. 2006. Comparison of five different immunoassays for the detection of Borrelia burgdorferi IgM and IgG antibodies. Clin Microbiol Infect 12:648–655. doi:10.1111/j.1469-0691.2006.01448.x 16774561

[49] Crother TR, Champion CI, Wu XY, Blanco DR, Miller JN, Lovett MA. 2003. Antigenic composition of Borrelia burgdorferi during infection of SCID mice. Infect Immun 71:3419–3428. doi:10.1128/IAI.71.6.3419-3428.2003 12761126 PMC155750

[50] Landry ML, Hassan S, Rottmann BG, Pesak SJ, Ordazzo M, Skrzyniarz M, Deponte S, Peaper DR. 2024. Performance of two modified two-tier algorithms for the serologic diagnosis of Lyme disease. J Clin Microbiol 62. doi:10.1128/jcm.00139-24 PMC11077974 38597655

[51] Arnaboldi PM, Sambir M, Dattwyler RJ. 2014. Decorin binding proteins A and B in the serodiagnosis of Lyme disease in North America. Clin Vaccine Immunol 21:1426–1436. doi:10.1128/CVI.00383-14 25121778 PMC4266351

[52] Signorino G, Arnaboldi PM, Petzke MM, Dattwyler RJ. 2014. Identification of OppA2 linear epitopes as serodiagnostic markers for Lyme disease. Clin Vaccine Immunol 21:704–711. doi:10.1128/CVI.00792-13 24623628 PMC4018895

[53] Radtke FA, Ramadoss N, Garro A, Bennett JE, Levas MN, Robinson WH, Nigrovic PA, Nigrovic LE, Pedi Lyme N. 2021. Serologic response to Borrelia antigens varies with clinical phenotype in children and young adults with Lyme disease. J Clin Microbiol 59:e0134421. doi:10.1128/JCM.01344-21 34379528 PMC8525570

[54] Joung H-A, Ballard ZS, Wu J, Tseng DK, Teshome H, Zhang L, Horn EJ, Arnaboldi PM, Dattwyler RJ, Garner OB, Di Carlo D, Ozcan A. 2020. Point-of-care serodiagnostic test for early-stage Lyme disease using a multiplexed paper-based immunoassay and machine learning. ACS Nano 14:229–240. doi:10.1021/acsnano.9b08151 31849225

[55] Ghosh R, Joung H-A, Goncharov A, Palanisamy B, Ngo K, Pejcinovic K, Krockenberger N, Horn EJ, Garner OB, Ghazal E, O’Kula A, Arnaboldi PM, Dattwyler RJ, Ozcan A, Di Carlo D. 2023. Single-tier point-of-care serodiagnosis of Lyme disease. bioRxiv. doi:10.1101/2023.06.14.544508 PMC11336255 39164226

[56] Lahey LJ, Panas MW, Mao R, Delanoy M, Flanagan JJ, Binder SR, Rebman AW, Montoya JG, Soloski MJ, Steere AC, Dattwyler RJ, Arnaboldi PM, Aucott JN, Robinson WH. 2015. Development of a multiantigen panel for improved detection of Borrelia burgdorferi infection in early Lyme disease. J Clin Microbiol 53:3834–3841. doi:10.1128/JCM.02111-15 26447113 PMC4652118

